# Origin of Gipsies

**Published:** 1874-03

**Authors:** 


### ORIGIN OF GIPSIES.

CHARLES LELAND, in his work on English Gipsies, speaks of the race of which they are a part as "the descendants of a vast number of Hindoos, of the primitive tribes of Hindoostan, who were expelled or migrated from that country early in the fourteenth century." The migration probably began earlier, for there are intimations of them as far west as Germany in 1416, and in 1427 a troop of them, numbering a hundred, appeared in Paris, where they gave themselves out as Christian Gipsies expelled from Egypt by the Mohammedans. No settled account of their origin is given by the Gipsies of any two lands in the old world, but their tradition tends on the whole towards the Egyptian origin which the popular notions of European nations had in general till of late assigned to them. Yet that the Rom or Romni are to be identified with the Dom or Domni caste of Hindoos, allied to the Nats, the real Gipsies of India at the present day—the letters D and R being hardly distinguishable in Gipsy mouths—is not only attested by the name they give themselves, but borne out by proofs without limit from the study of their speech and of their characteristic customs or habits.

